# Early oral feeding and its impact on postoperative outcomes in head and neck cancer surgery: a meta-analysis

**DOI:** 10.1186/s40902-024-00421-0

**Published:** 2024-03-28

**Authors:** Yomna E. Dean, Karam R. Motawea, Bdoor Ahmed A. Bamousa, Jose J. Loayza Pintado, Sameh Samir Elawady, Mohammed Soffar, Jaffer Shah, Kailyn Wilcox, Hani Aiash

**Affiliations:** 1 Faculty of Medicine, Alexandria University, Alexandria, Egypt; 2 Alfaisal University, Riyadh, Saudi Arabia; 3 Universidad de San Martin de Porres Facultad de Medicina Humana, La Molina, Peru; 4 Neuro-Endovascular Surgery Department, Medical University of South Carolina (MUSC), Charleston, USA; 5 Weill Cornell Medicine, New York, NY USA; 6 Division of Plastic Surgery, Tower Health Reading Hospital, Reading, USA; 7 SUNY Upstate Medical University, Syracuse, USA

**Keywords:** Free tissue flaps, Head and neck cancer, Reconstruction, Postoperative period

## Abstract

**Background:**

Early oral feeding has been previously postulated to contribute to developing postoperative complications following head and neck reconstructive surgeries using free flaps. This study assessed the association between the timing of oral feeding (early vs. late) and postoperative complications and length of hospital stay among these patients.

**Method:**

PubMed, Scopus, Cochrane, and Web of Science were searched using terms such as “oral feeding” and “head or neck cancer.” We utilized RevMan software version 5.4 for the analysis. The study defined early oral feeding as feeding within 5-day post-operation, while late oral feeding was defined as feeding after the fifth postoperative day. Five papers that met the inclusion criteria were included in the analysis, with 1097 patients.

**Results:**

The results showed that early feeding was not significantly associated with postoperative fistulas (*RR* 0.49, 95% *CI* 0.23 to 1.05, *p*-value = 0.07), hematoma/seroma (*RR* 0.71, 95% *CI* 0.33 to 1.51, *p*-value = 0.38), or flap failure (*RR* 0.84, 95% *CI* = 0.38 to 1.87, *p*-value = 0.67). However, early oral feeding was significantly associated with shorter hospital stays than late oral feeding (*MD* −3.18, 95% *CI* −4.90 to −1.46, *p*-value = 0.0003).

**Conclusion:**

No significant difference exists between early and late oral feeding regarding the risk of postoperative complications in head and neck cancer (HNC) patients who underwent free flap reconstruction surgery. However, early oral feeding is significantly associated with a shorter hospital stay than late oral feeding. Thus, surgeons should consider implementing early oral feeding after free flap reconstruction in HNC patients.

## Background

Head and neck cancers (HNC) were the seventh most common type of cancer worldwide in 2020. Oral and advanced laryngeal tumors are primarily treated by surgical excision, potentially necessitating free flap reconstruction [1, 2]. After being initially described more than four decades ago, free flap reconstruction has become widely acknowledged as the preferred method for reconstructing the majority of mucosal ablative defects in the head and neck region [3, 4]. Since vascularized free tissue transfer surgery has shown promise in many cases of head and neck reconstruction, where the surgical team achieves proficient speech, chewing, and swallowing functionality while also improving aesthetic results, free flap reconstruction has supplanted regional flaps and grafts [5]. Despite this, multiple postoperative complications such as fistula (incidence of around 5.8% [6]), seroma (incidence between 5 and 6% [7, 8]), wound/surgical site infection (incidence of up to 16.5% [9]), and flap failure (incidence of less than 3% [10]) still exist [11]. Accordingly, studies have aimed to identify several prognostic factors and protocols that could minimize such complications [12].

In the past, there was a belief that initiating oral feeding shortly after surgery or removing feeding tubes early following significant head and neck reconstruction led to heightened complications, including the development of orocutaneous or pharyngocutaneous fistulas and flap separation [13]. The reasoning behind this assumption was mainly attributed to the added stress on the suture lines imposed by the early utilization of the muscles [14]. And because of tradition, patients recovering from reconstructive oral surgery using a free flap are kept at NPO for 6–12 days [15, 16]. The consensus among experts regarding the perioperative care of individuals undergoing head and neck cancer-free tissue flap reconstruction, as outlined by the Society for Enhanced Recovery after Surgery in 2017 [17], suggests the prompt reinitiation of oral feeding following the surgical procedure. Although the advantages of early removal of nasogastric tubes have been established for patients treated for laryngeal and esophageal cancers [18–20], limited research is available for individuals with oral cancer.

Therefore, this study aimed to compile the evidence on the association between early initiation of oral feeding and postoperative complications (e.g., fistula formation, seroma development, and flap failure) and length of hospital stay following HNC reconstructive free-flap surgeries.

## Methods

### Search strategy

A literature search of the following databases (PubMed, Scopus, Cochrane, and Web of Science) on 30th August 2022 and 16th August 2023, using key terms such as (“oral feed*,” OR “enteral feed*,” OR “enteral nutrition”) AND (flap) AND (head OR neck OR cancer OR carcinoma OR neoplasm), was performed to identify relevant studies.

### Definition of early and late oral feeding

Early oral feeding is defined using a cutoff of 5 days [14]: patients who were fed 5 days or earlier postoperatively were allocated to the early feeding group, while patients who received oral feeding after 5 days postoperatively were assigned to the late feeding group. Patients started on fluids and then progressed to a soft diet.

### Inclusion and exclusion criteria

The studies were filtered based on the following criteria

#### Inclusion criteria

This study encompasses observational studies written in English that included case-control and cohort studies involving adult patients (≥ 18 years) undergoing HNC surgery and providing information on the duration of hospital stay and when oral feeding should begin postoperatively.

#### Exclusion criteria

Commentary, reviews, systematic reviews, meta-analyses, case reports, case series, or involving animal research were not included. Upon encountering duplicate studies, we determined to include the most recent studies with the greatest number of participants.

We have restricted the research population to HNC patients with free-flap repair to reduce analytical heterogeneity.

### Study selection

Two independent reviewers evaluated the studies based on our criteria. If a consensus cannot be reached, a third independent reviewer was consulted to settle the conflict.

### Data extraction and quality assessment

Two reviewers independently extracted the data from each study. To ensure accuracy, the data was then compared. If a consensus cannot be reached, a third independent reviewer was consulted to settle the conflict.

The following details were extracted from the eligible studies in order to create the baseline and summary data: the first author’s last name, the year the study was published, the study design, the number of participants, their age, their sex, the cancer site, the histopathology, the type of flap, the tracheostomy, and the conclusion.

For the outcomes data, the following information was extracted: incidence of postoperative fistula, flap dehiscence, flap failure, neck hematoma or seroma, wound infection, tracheostomy rate, postoperative chemotherapy/radiotherapy, and length of hospital stay among patients receiving early postoperative feeding and late postoperative feeding.

The quality of the included articles was assessed according to the Newcastle-Ottawa scale (NOS) [21], where a score of 7 or more was considered a high-quality paper. In contrast, a score of 6 or less was deemed low quality.

### Data analysis

The analysis was conducted using RevMan version 5.4, where continuous data as mean difference (MD) and dichotomous data were represented as risk ratio (RR), along with their respective 95% confidence interval (CI). If the data exhibited insignificant heterogeneity, a fixed-effect model was utilized. On the other hand, the random effect model was used in cases of significant heterogeneity (*p*-value less than 0.10 or *I* ^2^ > 50%). Furthermore, we utilized a leave-one-out test to solve the heterogeneity. Results were deemed significant at a *p*-value < 0.05.

### Definition of heterogeneity

It is the variation or diversity in outcomes among the studies included in the meta-analysis. It may be due to different factors, such as the characteristics of the participants, study designs, the methods of analysis, or other sources of bias [22].

## Results

**Fig. 1:**
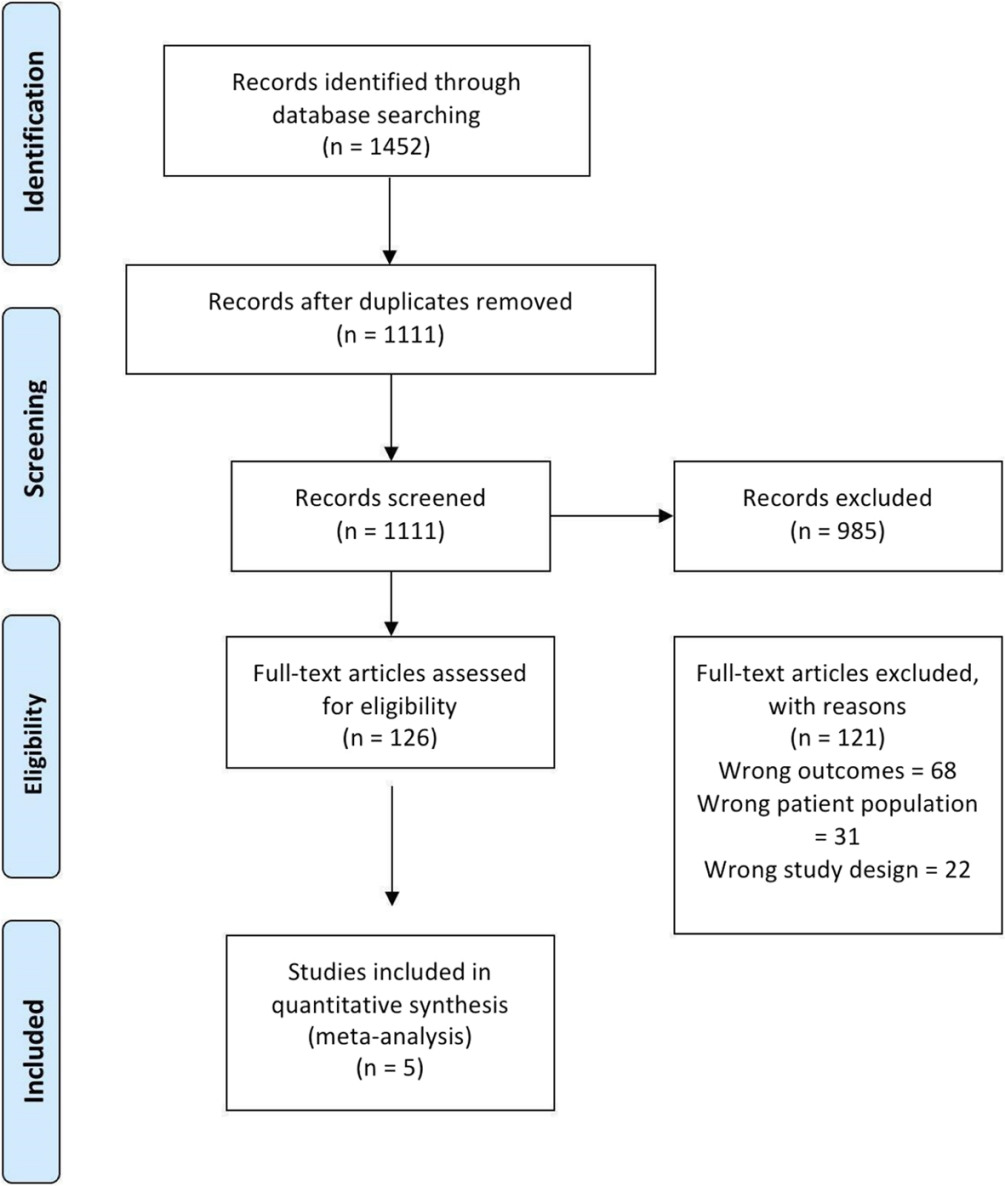
PRISMA flow diagram

A comprehensive search of the literature yielded 1434 studies. A total of 1093 studies were eligible for title and abstract screening after duplicates were eliminated. Then, 126 studies qualified for full-text screening, while 985 were deemed irrelevant. Ultimately, the meta-analysis included five studies [14, 23–26], as indicated by PRISMA [27] in Fig. 1.

A total of 1097 patients are included in the study: 384 patients who have undergone the early feeding protocol and 713 patients who have undergone the late feeding protocol. Table 1 contains further baseline data. Table 2 presents the quality rating of the listed research.

**Table 1:** Baseline and characteristics of the included studies

Author, year	Country	Study design	Sample size	Age, mean (SD)	Male, *N* (%)	Site of cancer	Type of the flap	Tracheostomy, patients (%)	Follow-up period in days Mean (SD)	Conclusion
Guidera (2013) [14]	New Zealand	Retrospective cohort	54	60.92 (21.07)		Mandible, maxilla, buccal mucosa, floor of mouth, hard palate, retromolar trigone, anterior 2/3rd of the tongue	Ulnar forearm, radial forearm, DCIA, and others	48 (88.9%) patients	14.72 (7.50)^a^	Early oral feeding is associated with shorter hospital stay and can improve postoperative outcome
Kerawala (2021) [23]	UK	Prospective cohort	400	62.15 (12.81)	228 (57%)		Fibula, DCIA, scapular donor sites, radial forearm free flap, anterolateral thigh flap	52 (13%) patients	16.1 (35.01)^a^	Early oral feeding is associated with shorter hospital stay and does not increase risk of perioperative complications
Stramiello (2021) [24]	United States	Retrospective cohort	104	62.29 (13.66)	68 (65.3%)	Site of H&N mucosal surgical defect:oral cavity, pharynx, larynx	Fibula, anterolateral thigh, osteocutaneous radial forearm free flap, fasciocutaneous radial forearm free flap		90	Early feeding does not increase risk of fistula and can improve swallowing function earlier
Le (2022) [25]	United States	Retrospective cohort	415	58.88 (14.18)	253 (61%)	Mandible, maxilla, tongue, buccal mucosa, floor of mouth	Radial forearm, osteocutaneous radial forearm, fibula, anterolateral thigh, DCIA	338 (81.4%)	761.53 (541.99)	Early oral feeding is safe and is associated with a decreased length of hospitalization
Wu (2022) [26]	China	Randomized controlled trial	128	51.5 (14.22)	76 (59.4%)	Buccal, maxilla Mandible, tongue, mouth floor, palate, roof of the tongue	Radial forearm, fibula, anterolateral femur, iliac crest	64 (50%)	30	Early oral feeding does not increase incidence of postoperative complications, and it is associated with minimizing pharyngeal pain and reduced hospital stay

^a^Follow-up period is the length of hospital stay

*Abbreviations*: *N* number, *SD* Standard deviation, *DCIA* Deep circumflex iliac artery

**Table 2:** Quality assessment of the included studies

	Selection				Comparability	Exposure			Total point
**Author (year)**	(1) Representativeness of the exposed cohort	(2) Selection of the nonexposed cohort	(3) Ascertainment of exposure	(4) Demonstration that outcome of interest was not present at start of study	(1) Comparability of cohorts on the basis of the design or analysis	(1) Assessment of outcome	(2) Was follow-up long enough for outcomes to occur	(3) Adequacy of follow-up of cohorts	
**Guidera (2013) **[14]	1	1	1	1	0	1	1	1	7
**Kerawala (2021) **[23]	0	1	1	0	2	1	1	1	7
**Stramiello (2021) **[24]	0	1	1	1	2	1	1	NA	7
**Le (2022) **[25]	1	1	1	0	2	1	1	1	8
**Wu (2022) **[26]	1	1	1	1	2	1	1	1	9

### Outcomes

#### Fistula

**Fig. 2:**
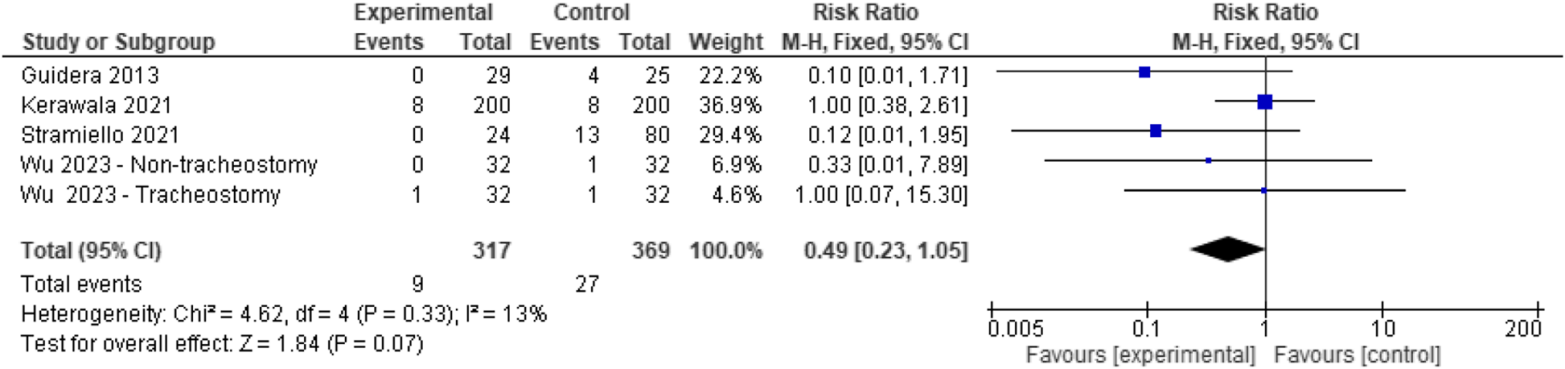
Forest plot showing the association between early oral feeding and fistula formation compared to the delayed feeding group

Early oral feeding led to a lower risk of postoperative fistulization (*RR* 0.49, 95% *CI* 0.23–1.05). Nevertheless, it was deemed insignificant at a *p*-value of 0.07. No heterogeneity was detected in the analysis (*p* = 0.33, *I* ^2^ = 13%) (Fig. 2).

#### Flap dehiscence and failure

**Fig. 3:**
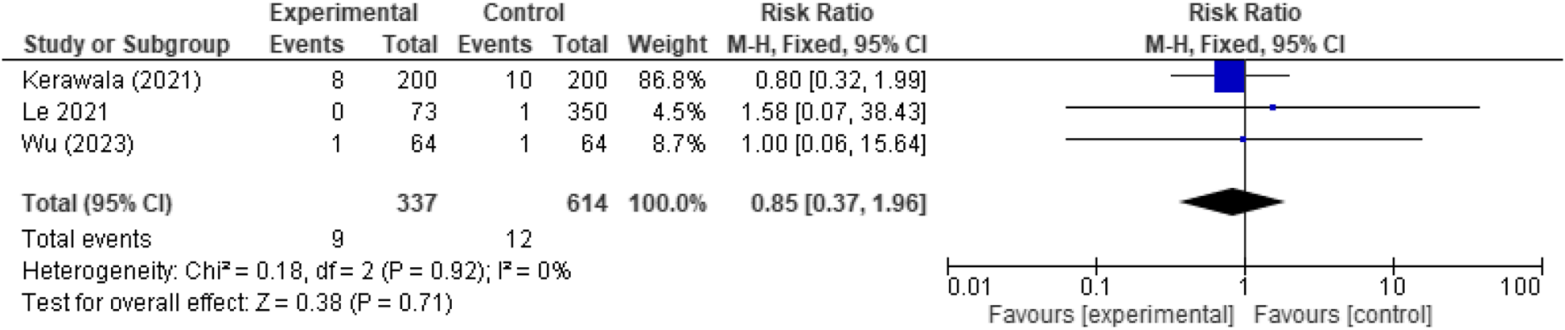
Forest plot showing the association between early oral feeding and flap dehiscence formation compared to the delayed feeding group

**Fig. 4:**
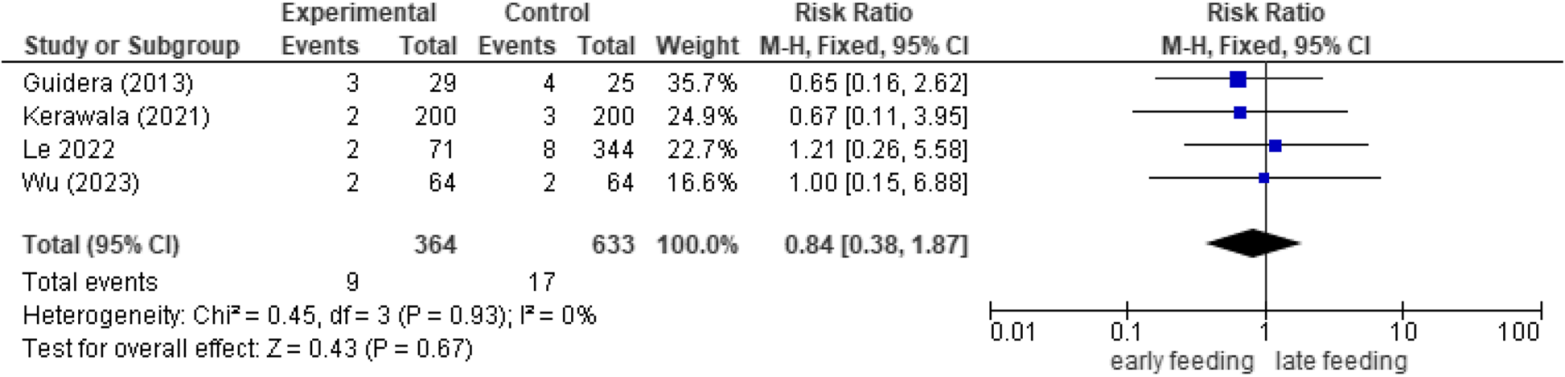
Forest plot showing the association between early oral feeding and flap failure compared to the delayed feeding group

No significant relation (*p*-value 0.71) was seen between the timing of feeding and rates of postoperative dehiscence and flap failure (*RR* 0.85, 95% *CI* 0.37 to 1.96, *p*-value 0.71, *RR* 0.85, 95% *CI* 0.37 to 1.96, *p*-value 0.67, respectively). No heterogeneity was detected in the analysis as well (Figs. 3 and 4).

#### Neck hematoma/seroma

**Fig. 5:**
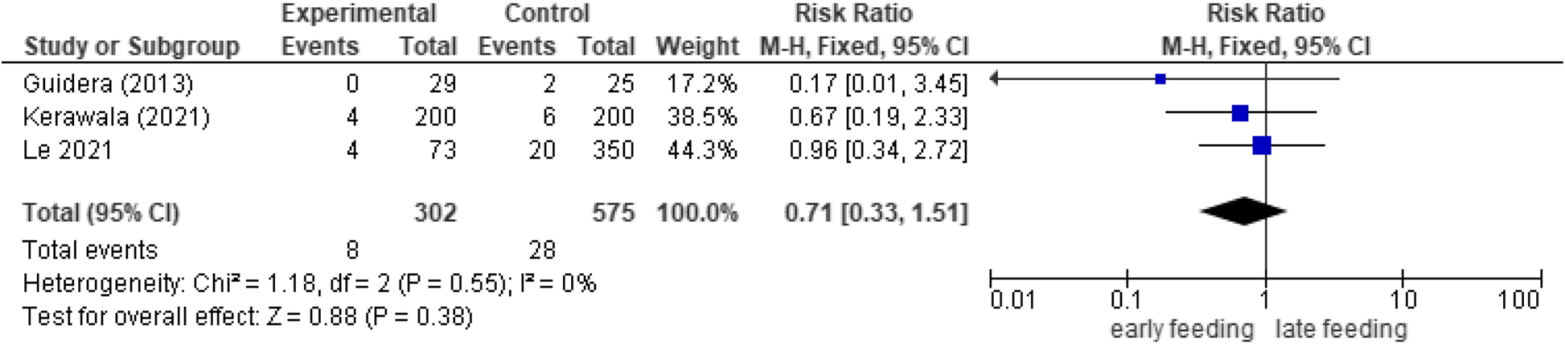
Forest plot showing the association between early oral feeding and seroma/hematoma formation compared to the delayed feeding group

There was not a significant association between the timing of oral feeding and the incidence of postoperative neck hematoma/seroma (*RR* 0.71, 95% *CI* 0.33 to 1.51, *p*-value 0.38). No heterogeneity was detected in the analysis (*p* = 0.55, *I* ^2^ = 0%) (Fig. 5).

#### Wound infection

**Fig. 6:**
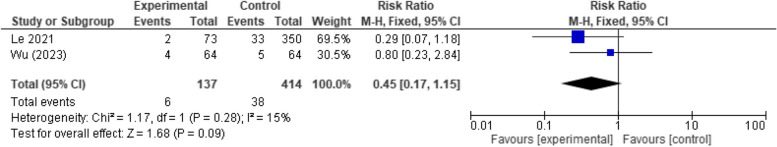
Forest plot showing the association between early oral feeding and wound infection compared to the delayed feeding group

There was not a significant association between the timing of oral feeding and the incidence of postoperative wound infection (*RR* 0.45, 95% *CI* 0.17 to 1.15, *p*-value 0.09). No heterogeneity was detected in the analysis (*p* = 0.28, *I* ^2^ = 15%) (Fig. 6).

#### Tracheostomy rate

**Fig. 7:**
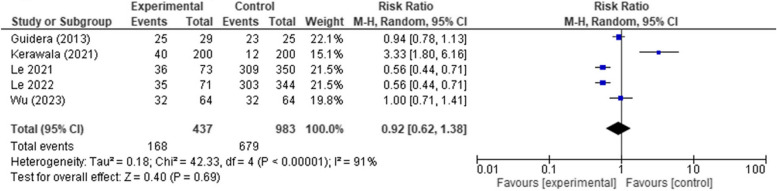
Forest plot showing the association between early oral feeding and tracheostomy rate compared to the delayed feeding group

There was not a significant difference between the early and late oral feeding regarding the tracheostomy rates (*RR* 0.92, 95% *CI* 0.62 to 1.38, *p*-value 0.69). Considerable heterogeneity was detected in the analysis that was not solved using the leave-one-out test (*p* < 0.00001, *I* ^2^ = 91%) (Fig. 7).

#### Preoperative chemotherapy/radiotherapy

**Fig. 8:**
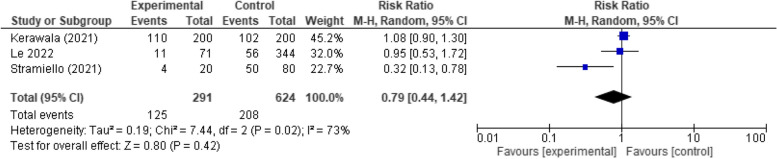
Forest plot showing the association between early oral feeding and preoperative radiotherapy/chemotherapy compared to the delayed feeding group

There was not a significant difference between the early and late oral feeding regarding the preoperative chemotherapy/radiotherapy administered (*RR* 0.79, 95% *CI* 0. 44 to 1.42, *p*-value 0.42). A considerable heterogeneity was detected in the analysis that was not solved using the leave-one-out test (*p* = 0.02, *I* ^2^ = 73%). As a result, we employed the leave-one-out test, where we removed Stramiello (2021), resolving the heterogeneity in the analysis (*p* = 0.69, *I* ^2^ = 0%). Nevertheless, the difference between both groups remained insignificant (*RR* 1.07, 95% *CI* 0.89 to 1.27, *p*-value 0.47) (Fig. 8).

#### Length of hospital stay (days)

**Fig. 9:**
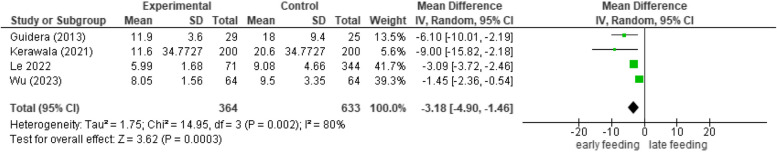
Forest plot showing the association between early oral feeding and length of hospital stay (days) compared to the delayed feeding group

The analysis demonstrated a significant decrease in the hospital duration among the early feeding group compared to late feeding (*MD* −3.18, 95% *CI* −4.90 to −1.46, *p*-value 0.0003). However, the analysis revealed significant heterogeneity (*p* = 0.002, *I* ^2^ = 80%). Subsequently, a leave-one-out test was employed where we removed Wu (2023) and, thus, resolved the heterogeneity. The resulting association demonstrated a further decline in the length of the hospital stay among the early feeding group (*MD* −4.91, 95% *CI* −8.06 to −1.76, *p*-value 0.002) (Fig. 9).

## Discussion

While there exists a theoretical concern regarding the potential for early feeding to disrupt mucosal sutures or increase the risk of wound contamination, we did not detect a statistical significance between early and late feeding in regard to the postoperative incidence of fistulas, strictures, hematoma/seromas, and flap failure rate. Furthermore, we conducted an analysis considering factors such as tracheostomy rate and preoperative chemotherapy/radiotherapy, which could influence the outcomes. Yet, no statistically noteworthy differences were evident between the early and late feeding cohorts. However, another notable finding was the length of hospital stay, which was significantly lower among patients receiving early postoperative nutrition.

One of the studies included in our analysis is Guidera et al. [14], which demonstrated no significant disparities in total complication numbers or local complications such as fistula. This challenges the conventional practice of postponing oral feeding after oral cavity cancer resection and reconstruction. Additionally, their research revealed a shorter hospital stay duration for the early feeding group, mirroring our findings and underscoring the potential drawbacks, be they physical, psychological, or financial, associated with delayed oral intake.

The results of our study indicate that early feeding does not raise the risk of postoperative complications. This aligns with the findings of McAuley et al. [28]; in this comparative study, oral cancer patients received a pureed diet on the fifth day postoperatively and earlier. No postoperative complications were detected among these patients, including orocutaneous fistula. Additionally, these patients reported decreased length of hospital stay, reinforcing our findings. Another study by Poisson et al. [29] concluded that early oral feeding is associated with a reduced incidence of postoperative complications among oral squamous cell carcinoma patients. Their analysis detected a significant association between non-oral feeding at the end of the hospital stay and major and minor surgical complications. This demonstrates a stark contrast from our data, further highlighting our finding that early feeding does not lead to an increased incidence of fistulas compared to late feeding, which is a major complication post reconstructive surgeries [30, 31]. Correspondingly, Kerawala et al. [23] demonstrated that early feeding after oral reconstruction with free tissue transfer did not lead to a heightened rate of local postoperative complications; more precisely, there were no increased rates of flap dehiscence.

Brady et al. [32] demonstrated that early oral feeding was associated with a significant reduction in the length of hospital stay, with a median length of 10 days in comparison to previous studies conducted at the same center where patients received delayed feeding and subsequently had a longer hospital stay, with a median length of 20 days. A recent study conducted by Le et al. [25] produced similar findings; 415 patients were included, with 71 patients allocated to the early feeding group (< 5 days) and 344 patients allocated to the late feeding group (> 5 days). Their analysis revealed that early feeding was associated with fewer postoperative complications and a shorter hospital stay. Furthermore, in a study with a small sample size, Stramiello et al. [24] identified a statistically significant difference in fistula incidence between early and late feeding groups, favoring the early feeding approach.

A recent meta-analysis focusing on the risk of postoperative fistula among HNC patients concluded that the timing of oral feeding does not affect fistulization rates, regardless of preoperative chemoradiotherapy, which might hinder wound healing [33].

In another study, a randomized clinical trial conducted by Wu et al. [26], it was demonstrated that oral cancer patients offered early oral intake postoperatively did not suffer from increased incidence of wound complications and pneumonia. In the same clinical trial, patients who had a tracheostomy and were assigned to the early feeding group had decreased pharyngeal pain and a shortened hospital stay compared with patients who had tracheostomy and delayed oral feeding.

### Strength and limitations

To the best of our knowledge, this is the first meta-analysis addressing the impact of early oral feeding on various postoperative complications as well as the duration of hospital stay following flap reconstruction in head and neck cancer patients. We integrated similar studies, resulting in minimal heterogeneity within our analysis. However, our study population was limited to 1520 patients due to the scarcity of published papers on post-free flap reconstruction oral feeding timing in head and neck cancer patients. Additionally, the average age of the patients included was nearly 60, primarily focusing on the elderly population, which reflects the higher prevalence of head and neck cancer in this demographic. Future research should encompass various age groups, cancer site locations, histopathology, free-flap types, and postoperative complication incidences to investigate the optimal timing for postoperative oral feeding comprehensively.

## Conclusions

No significant difference exists between early and late oral feeding regarding the risk of postoperative complications in HNC patients who underwent free flap reconstruction surgery. However, early oral feeding is significantly associated with shorter length of hospital stay compared to late oral feeding. Accordingly, surgeons should consider implementing early oral feeding protocol after free flap reconstruction among HNC patients.

## Data Availability

Data is available upon request to the corresponding author.

## Abbreviations

HNC: Head and neck cancer
EF: Early oral feeding
LF: Late oral feeding
NPO: Nothing by mouth
NOS: Newcastle-Ottawa scale
MD: Mean difference
CI: Confidence interval
RR: Relative ratio
